# A large proportion of asymptomatic *Plasmodium *infections with low and sub-microscopic parasite densities in the low transmission setting of Temotu Province, Solomon Islands: challenges for malaria diagnostics in an elimination setting

**DOI:** 10.1186/1475-2875-9-254

**Published:** 2010-09-07

**Authors:** Ivor Harris, Wesley W Sharrock, Lisa M Bain, Karen-Ann Gray, Albino Bobogare, Leonard Boaz, Ken Lilley, Darren Krause, Andrew Vallely, Marie-Louise Johnson, Michelle L Gatton, G Dennis Shanks, Qin Cheng

**Affiliations:** 1 Australian Army Malaria Institute, Weary Dunlop Drive, Gallipoli Barracks Enoggera, Qld 4051, Australia; 2 Malaria Drug Resistance and Chemotherapy, Queensland Institute of Medical Research, Brisbane, Australia; 3 Pacific Malaria Initiative Support Centre, School of Population Health, University of Queensland, Brisbane, Australia; 4 Vector Borne Disease Control Programme, Ministry of Health, Solomon Islands

## Abstract

**Background:**

Many countries are scaling up malaria interventions towards elimination. This transition changes demands on malaria diagnostics from diagnosing ill patients to detecting parasites in all carriers including asymptomatic infections and infections with low parasite densities. Detection methods suitable to local malaria epidemiology must be selected prior to transitioning a malaria control programme to elimination. A baseline malaria survey conducted in Temotu Province, Solomon Islands in late 2008, as the first step in a provincial malaria elimination programme, provided malaria epidemiology data and an opportunity to assess how well different diagnostic methods performed in this setting.

**Methods:**

During the survey, 9,491 blood samples were collected and examined by microscopy for *Plasmodium *species and density, with a subset also examined by polymerase chain reaction (PCR) and rapid diagnostic tests (RDTs). The performances of these diagnostic methods were compared.

**Results:**

A total of 256 samples were positive by microscopy, giving a point prevalence of 2.7%. The species distribution was 17.5% *Plasmodium falciparum *and 82.4% *Plasmodium vivax*. In this low transmission setting, only 17.8% of the *P. falciparum *and 2.9% of *P. vivax *infected subjects were febrile (≥38°C) at the time of the survey. A significant proportion of infections detected by microscopy, 40% and 65.6% for *P. falciparum *and *P. vivax *respectively, had parasite density below 100/μL. There was an age correlation for the proportion of parasite density below 100/μL for *P. vivax *infections, but not for *P. falciparum *infections. PCR detected substantially more infections than microscopy (point prevalence of 8.71%), indicating a large number of subjects had sub-microscopic parasitemia. The concordance between PCR and microscopy in detecting single species was greater for *P. vivax *(135/162) compared to *P. falciparum *(36/118). The malaria RDT detected the 12 microscopy and PCR positive *P. falciparum*, but failed to detect 12/13 microscopy and PCR positive *P. vivax *infections.

**Conclusion:**

Asymptomatic malaria infections and infections with low and sub-microscopic parasite densities are highly prevalent in Temotu province where malaria transmission is low. This presents a challenge for elimination since the large proportion of the parasite reservoir will not be detected by standard active and passive case detection. Therefore effective mass screening and treatment campaigns will most likely need more sensitive assays such as a field deployable molecular based assay.

## Background

Accurate and timely diagnosis of malaria infections is a critical part of case management in malaria control programmes aiming to reduce malaria morbidity and mortality. Sensitive and effective detection of infected individuals also plays a vital role in areas where transmission has been reduced markedly due to intensified control measures and elimination is being considered.

Malaria diagnosis is traditionally achieved by microscopic examination of blood smears. Microscopy is able to detect parasite species and determine parasite densities. The limit of detection by thick film microscopy is in the range of 5 to 100 parasites/μL of blood [1-3]. However, the quality of microscopy can vary significantly [1,4] because its accuracy largely relies on the experience and training of the microscopists to make and stain a blood slide correctly and read it accurately.

Malaria rapid diagnostic tests (RDTs) have been developed and tested over the past 2 decades as an alternative to microscopy, particularly for areas where quality microscopy is absent or hard to maintain. RDTs are lateral flow devices that detect parasite proteins using antibodies. The tests are easy to perform and provide rapid results in 15 to 20 minutes without the need for electricity, expensive equipment or extensive training. The limit of detection, by good quality RDTs, is similar to that of thick film microscopy for *P. falciparum *but often poorer for *P. vivax *[1]. Today, over 50 brands of malaria RDTs are manufactured, and over 150 individual products commercially available.

Polymerase chain reaction (PCR) is a DNA-based molecular detection method that is more sensitive than microscopy and RDT, and has been widely used for diagnosis, confirmation of diagnosis, epidemiology studies and drug efficacy assessment. In theory, PCR is capable of detecting a single parasite in a blood sample, and its sensitivity often is only limited by the volume of the blood. PCR provides accurate determination of parasite species, better sensitivity in detecting low density of parasites and better detection of mixed species/strain infections [2,5,6]. In the field, PCR has been mostly used in epidemiological studies instead of point of care diagnosis due to its requirement for sophisticated equipment, reagents and several hours of turn over time.

In most malaria control settings, where the goal is to significantly reduce malaria morbidity and mortality, quality assured microscopy and RDTs have been shown to effectively detect parasites in the majority of symptomatic patients and thus guide treatment. In contrast, for malaria elimination settings it is critical to detect all infections, including those with low and sub-microscopic parasite densities in asymptomatic carriers as they represent a parasite reservoir in the community capable of effectively transmitting infections to mosquitoes [7,8] and seeding transmission foci [9].

It is well known that malaria epidemiology varies between country and region, and particularly between islands, because the dominant vector species, the characteristics of human populations and factors that influence transmission such as rainfall, temperature, housing conditions and population movement differ. Therefore, the challenges to malaria elimination in different settings will vary. Each area needs to investigate the malaria epidemiology and carefully tailor its diagnosis strategy to the local context.

Temotu Province in Solomon Islands is preparing for malaria elimination. An assessment of the epidemiological characteristics of malaria infections in the island population, particularly the prevalence and distribution of asymptomatic infections, and infections with low or sub-microscopic parasite densities will contribute to the understanding of the requirements of diagnostics in malaria elimination. A baseline malaria parasitological survey was conducted in Temotu Province in October-November 2008 as the first step in a provincial malaria elimination programme. This baseline survey provided opportunities to obtain point prevalence and epidemiological characteristics of malaria infections, as well as to assess how well different diagnostic methods performed in this particular setting. During the survey, blood samples collected from the population were examined by microscopy, with a subsample examined by PCR and RDTs. The overall malaria point prevalence was estimated at 2-3% with predominant *P. vivax *infections [10]. Of concern for diagnosis was the high prevalence of asymptomatic infections (86%) reported in this relatively low transmission setting.

To better understand malaria epidemiology in the Province, the basis of the observed low disease rate and to better evaluate the performance of different diagnostic methods, a detailed comparison of the microscopy, RDT and PCR was undertaken, including re-examination of the blood smears from discrepant samples and the determination of parasite densities. The results revealed that a large proportion of malaria infections in the province were at low and sub-microscopic parasite densities. The finding has an important practical implication to the malaria elimination strategy in the province.

## Methods

### Survey site and sample collection

The survey was conducted by the Pacific Malaria Initiative Survey team in the Temotu Province, a remote island group in the Solomon Islands, approximately 166°E, 11°S with a population of approximately 17,000. The survey site, consent process and sample collection methods are as previously described [10].

### Preparation of blood films, microscopic examination and quality assurance

Thick and thin blood films from finger prick blood of 9,491 survey participants were prepared, air dried, stained and examined as reported earlier [10]. All slides which were considered positive on the initial examination, negative on the initial examination but positive by PCR and a 10% sample of initial microscopy negative samples, were subjected to a second quality assurance (QA) examination (of 300 fields in the thick film). All slides which recorded discrepant results between the initial examination and the QA examination were then subjected to a third "referee" examination (of 500 fields in the thick film), which was recorded as the final microscopy result. Identification of parasite species was performed by reference to the thin smears where parasites densities were sufficiently high enough. Otherwise, parasite species identification was made by reference to the thick smear only.

Malaria microscopists who participated in this survey were all certified to WHO malaria microscopy standards by prior participation and assessment in WHO accredited malaria microscopy competency assessment courses in Brisbane or Honiara. Microscopists who performed the initial slide examinations were all certified to competency levels 1, 2 or 3. Microscopists who performed the QA examination were all certified to 1 or 2, and microscopists who performed the third "referee" examinations were certified to competency level 1.

### Parasite density counts

Parasite densities were determined by counting the number of parasites against 500 white blood cells (WBC) and calculated assuming 8,000 WBC per μL of blood.

### PCR assays

Genomic DNA was extracted from a subset of 1,784 filter paper samples. These included samples that were microscopy positive, samples from all febrile patients and 10% of the microscopy negative samples from each village. Following microscopy QA, some of the samples initially included as microscopy positive were reclassified as microscopy negative. This increased the percentage of negative samples examined to 15.9%. The DNA was extracted using QIAamp DNA Mini Kits and a QIAcube robot (QIAGEN, Crawley, UK), and eluted to a 100 μl volume. A multiplex PCR was performed to determine parasite species using published primers and PCR conditions [11] with the number of cycles increased from 43 to 45. The positive results were confirmed using a second round *P. falciparum *and *P. vivax *specific PCR.

### RDTs

ICT Malaria Combo Cassette Test ML02 (ICT diagnostics) was used as per the manufacturer's instructions when a participant was febrile and on a subset of 400 children from villages around Lata, the Provincial capital. For QA, a post purchasing sample of these RDTs were tested at the Research Institute for Tropical Medicine, Manila, Philippines, a WHO-FIND lot testing laboratory and were found suitable. All RDTs were used within one month of the commencement of the survey.

## Results

### Malaria prevalence determined by microscopy

A total of 9,491 blood samples were collected and examined by microscopy. After the initial microscopy and two rounds of microscopy QA, 256 samples were determined positive giving an overall point prevalence of 2.7%. *P. falciparum *and *P. vivax *made up 17.5% (45/256) and 82.4% (211/256) of positive samples respectively. Two of the 256 positive samples were excluded from density related analyses because parasite densities were not determined due to poor quality of the slides.

### A low percentage of microscopy positive subjects were symptomatic

The proportion of microscopy positive individuals having an aural temperature ≥38°C at the time of examination was 17.8% (8/45) for *P. falciparum *and 2.9% (6/209) for *P. vivax*, respectively, giving an overall fever rate of 5.5% (14/254). The majority of fevers, 50.0% (4/8) and 66.7% (4/6) for *P. falciparum *and *P. vivax*, were observed in the 5-14 year age group. Parasite densities in six of the eight *P. falciparum*-infected fever subjects were >500/μL. In contrast, 66.7% of *P. vivax *infected fever subjects had parasite densities <100/ μL.

### A large proportion of microscopy positive subjects had low parasite densities

**Figure 1:**
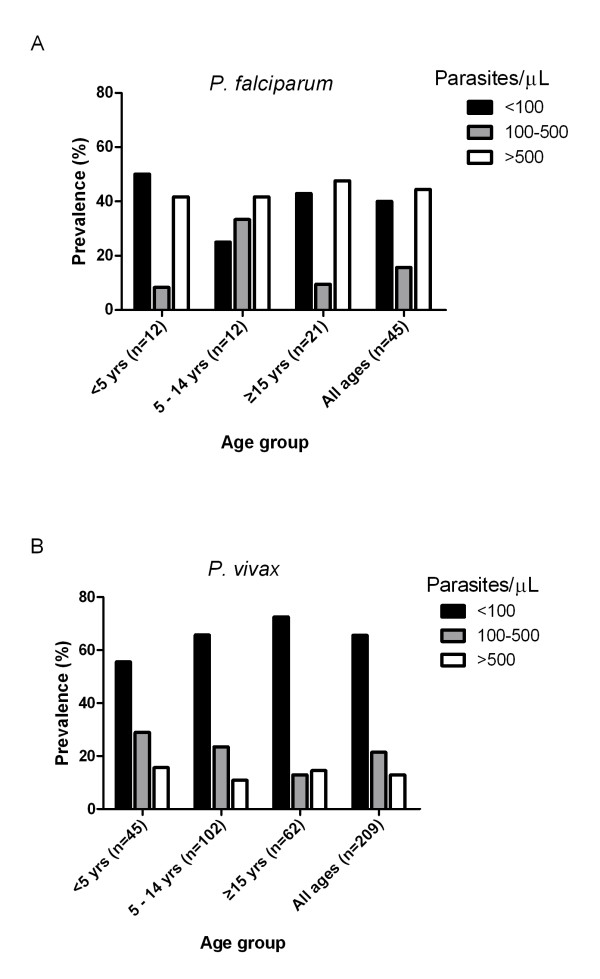
**Proportion of microscopy positive *P. falciparum *(1A) and *P. vivax *(1B) individuals of different age groups having parasite densities <100, 100 -500 and >500 parasite/μL**.

Parasite densities were determined for 254 of the 256 microscopy positive subjects and divided into three groups: <100, 100-500 and >500/μL. Overall, 61.0% (155/254) of these subjects, including 40.0% (18/45) of the *P. falciparum *and 65.6% (137/209) of the *P. vivax *infected subjects, had parasite densities < 100/μL (Figure 1). The proportion of subjects with parasite densities < 100/μL increased with age for *P. vivax *infections: 55.6% in the < 5 year-old group and reaching 72.6% in the ≥15 year-old age group (Figure 1B). No age correlation was observed for the proportion of *P. falciparum *infections having low parasite densities (Figure 1A).

### A large number of sub-microscopic infections were detected by PCR

PCR was performed on a subset of 1,784 samples including microscopy positive (n = 256), febrile but microscopy negative (n = 59) and microscopy negative (n = 1469). Of the 256 microscopy positive samples, PCR detected *Plasmodium *DNA in 223 samples, including 55 (24.7%) *P. falciparum*, 139 (62.3%) *P. vivax *and 29 (13.0%) mixed *P. falciparum-P. vivax*. In the subset of 1,469 microscopy negative samples that were randomly selected from different villages, PCR detected 63 *P. falciparum*, 23 *P. vivax *and 10 mixed infections. These sub-microscopic infections, representing 18.6% of all observed infections, were distributed between 33 of the 43 villages where malaria infections were identified.

### Malaria prevalence estimated by PCR

**Figure 2:**
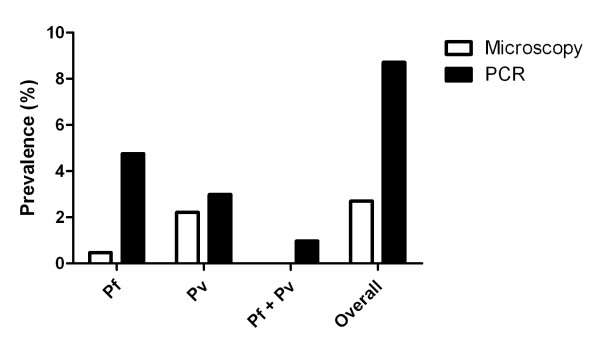
**Parasite prevalence determined by microscopy and estimated by PCR**.

Assuming that the PCR positive rate (96/1,469) applies to the entire microscopy negative group, 603.5 samples would be expected to be PCR positive in 9,235 samples. Adding the 223 samples positive by both microscopy and PCR, the total number of PCR positive samples in 9,491 samples would be 826.5, giving an overall parasite prevalence of 8.7%, 3.3 fold higher than that determined by microscopy (Figure 2). The expected species distribution for *P. falciparum*, *P. vivax *and mixed infections would be 4.8%, 3.0% and 1.0%, respectively (Figure 2).

### Comparison of microscopy and PCR

**Figure 3:**
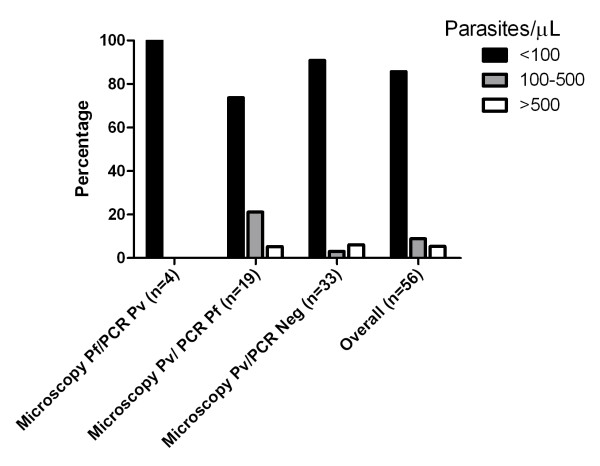
**Distribution of parasite densities in microscopy and PCR discrepant samples**.

A total of 1,784 samples were examined by both microscopy and PCR producing 352 samples that were positive by either or both methods (Table 1). In this subset, there was an 85.9% concordance in speciation between methods. For samples that were PCR positive for a single species (*P. falciparum *or *P. vivax*) there was greater concordance with microscopy results for *P. vivax *(135/162) compared to *P. falciparum *(36/118) (Table 1). Ignoring samples identified as mixed infections by PCR, 85.7% of the discordant samples had parasite densities below 100/μL (Figure 3). PCR detected 63 *P. falciparum*, 23 *P. vivax *and 10 mixed infections in 1,469 microscopy negative samples, suggesting that parasite densities in these samples are at sub-microscopy level.

**Table 1:** Comparison of a subset of samples examined by both microscopy and PCR (n = 1784).

	Microscopy					
		**Pf**	**Pv**	**Pf + Pv**	**Neg**	

PCR	Pf	36	19	0	63	118
	
	Pv	4	135	0	23	162
	
	Pf + Pv	5	24	0	10	39
	
	Neg	0	33	0	1432	1465
	
	Total	45	211	0	1528	1784

The 33 microscopy positive but PCR negative samples were examined by two level 1 (expert) microscopists and determined as *P. vivax*. Of these, 31 (94.0%) had parasite densities <100/μL, of which 18 samples were counted as 8-10/μL, which is equivalent to one parasite in 100-300 fields on a thick film.

### Performance of RDT

ICT malaria combo tests were used to test 414 subjects. The RDT detected 25 *P. falciparum *and one *P. vivax *infection. The ICT combo test detected all 12 microscopy and PCR confirmed *P. falciparum *infections, including two samples with parasite densities <100/μL (Table 2). The tests also returned positive *P. falciparum *results on 10 microscopy and PCR negative samples. In contrast, it only detected one of the 13 microscopy and PCR confirmed *P. vivax *samples; this sample had a parasite density >500/μL (Table 2). The 12 *P. vivax *samples that the ICT combo test failed to detect had parasite densities either <100/μL (n = 6) or 100-500/μL (n = 6) (Table 2).

**Table 2:** Comparison of detection by ICT malaria combo tests and microscopy, PCR

RDT	Parasite density (P/μL)	Microscopy results/PCR results								
		
		Pf/Pf	Pv/Pv	Pf/Pf+Pv	Pv/Pf	Neg/Pf	Neg/Pv	Neg/Pf+Pv	Neg/Neg	Total
RDT Pos	**Total**	**10**	2*	**2**	**1**	**1**	**0**	**0**	**10**	**26**
	
	< 100	0	0	1	1	-	-	-	-	2
	
	100-500	2	0	0	0	-	-	-	-	2
	
	> 500	8	2	1	0	-	-	-	-	11

RDT Neg	**Total**	**1**	**12**	**0**	**0**	**12**	**5**	**1**	**357**	**388**
	
	< 100	1	6	-	-	-	-	-	-	7
	100-500	0	6	-	-	-	-	-	-	6
	> 500	0	0	-	-	-	-	-	-	0

Note:* RDT determined 1 positive for *P. falciparum *and 1 positive for *P. vivax*.

## Discussion

Temotu Province has been selected as a possible malaria elimination site based on the reduction of reported malaria cases due to past malaria control measures. The mass blood survey conducted in November 2008 showed that the overall malaria prevalence at the time of survey, based on microscopy was 2.7%, indicating the Province as a low transmission area.

Based on the low transmission rate determined by microscopy, it would be expected that the level of acquired immunity in the community would be relatively low, and consequently the proportion of infected subjects with symptoms and high parasite densities would be high. The survey findings were contrary to this expectation. Firstly, only 5.5% of the microscopy positive individuals had fever at the time of survey. Secondly, 61.0% of microscopy positive individuals had parasite densities below 100/μL. Such a high proportion of subjects having low parasite densities was reported recently in an area of Cambodia where *P. falciparum *prevalence was >40% [12], reflecting a high level of acquired immunity in the community. Observations from the current study show the proportion of *P. vivax *infected subjects with parasite densities <100/μL increased with age, suggesting a correlation with exposure and acquired immunity. However, how immunity is maintained in an area with such low transmission is unclear. Other factors such as host genetics and parasite genetics may also be involved in determining the epidemiological situation observed.

The microscopy results suggest that in Temotu Province a large proportion of individuals infected with *Plasmodium *have parasite densities at the limit of microscopy and RDT detection. The addition of PCR testing of samples enhances the detection capability. Compared to PCR, microscopy performed better in detecting *P. vivax *than detecting *P. falciparum *infected subjects as the *P. vivax *prevalence determined by microscopy was 73% of that estimated by PCR, higher than the average of 50% reported from a meta-analysis of 72 pairs of prevalence measurements [13], while the microscopy determined *P. falciparum *prevalence was only 9.7% of that estimated by PCR. It should be noted that the microscopy results obtained here were by WHO certified level 1 microscopists. This level of detection and accuracy would not have been achieved in many aid posts and health centres which often have less experienced microscopists and thus the typical error in microscopy results could be greater than found here [1,14,15].

The PCR-estimated overall malaria prevalence was 8.7%, 3.3 fold higher than that estimated by microscopy alone. This suggests that there is a substantial proportion of the *Plasmodium *infected subjects infected with sub-microscopic parasite densities, in addition to the 61% of subjects with low parasite densities detected by microscopy. The number of sub-microscopic infections is likely higher than reported since PCR itself has a limit of detection. This limit may be decreased if a PCR targets a gene with higher copy numbers compared to the ssrRNA gene used in this study. Interestingly, the sub-microscopic infections were distributed across the island in 32 of the 43 villages where malaria infections were identified. The findings are in agreement with the result of a meta-analysis demonstrating the proportion of sub-microscopic infection of *P. falciparum *is significantly higher in areas of low transmission [13]. These sub-microscopic and low density infections may persist for a period of time without causing any symptoms and, therefore, contribute to the maintenance of acquired immunity.

PCR is expected to generally have a higher sensitivity than microscopy in detecting very low parasite density infections as well as detecting mixed infections. Interestingly, the majority of samples that were PCR positive and microscopy negative were identified by PCR as *P. falciparum*. An almost identical finding was reported recently from Cambodia [4] where microscopy detected a total of 350 *P. falciparum *infections while PCR detected a further 331 *P. falciparum *single infections from microscopy negative samples. A possible explanation for this discrepancy is that in *P. falciparum *usually only ring-stage parasites are present in blood smears and they are smaller than the ring-stage and mature trophozoites of *P. vivax *and may be easier to miss by microscopy particularly when staining is less than perfect.

There were also discrepancies between microscopy and PCR in determining the parasite species. All four microscopy *P. falciparum*/PCR *P. vivax *samples and 14 of the 19 microscopy *P. vivax*/PCR *P. falciparum *samples had parasite densities <100/μL. In these low parasite density infections microscopic species identification is based on the appearance of only one or two parasites in a thick smear, and therefore their accuracy may be less than optimal. Additionally, some of these discrepancies may be caused by mixed species infections that had low density parasites of both species and were detected by microscopy as one and PCR as another species.

PCR failed to detect 33 microscopy positive samples all of which were determined as *P. vivax *by microscopy. Of these, 94% had parasite densities <100/μL, and 18 samples were counted as 8-10/μL. At such a low counts, which is equivalent to one parasite in 100-300 fields on a thick film, the microscopy determination of *P. vivax *was made based on just one or a few mature trophozoites found on the thick smear. The reliability of microscopy results based on one or two parasites may be questionable. Alternatively, it is possible that this level of parasite density is also at the detection limit of PCR. Theoretically, the threshold of detection for PCR is limited to the presence of at least one parasite in a sample. At low parasite densities, the presence of a parasite in a small volume of blood is subject to stochastic variation. This, compounded with possible DNA degradation during storage and transportation of the samples, could result in a false negative PCR although this has been reported to be an unlikely cause [16]. Nevertheless, PCR returned positive results for 82/132 of the subjects with *P. vivax *densities <100/μL.

RDTs are playing an increasing role in malaria control and elimination. The ICT combo kit detects *P. falciparum *histidine rich protein 2(HRP2) and a *Plasmodium *pan-species antigen, aldolase. The performance of the *P. falciparum *detection component of the ICT combo kit was comparable to our level1 microscopists in detecting *P. falciparum *infections. The kit detected all but one microscopy positive *P. falciparum *sample, including two samples with parasite densities <100/μL. It failed to detect one *P. falciparum *sample with parasite density of 64/μL and 12 microscopy negative but PCR positive samples. ICT combo kit also determined 10 samples which were negative by both microscopy and PCR to be *P. falciparum *positive. This could be due to its detection of circulating PfHRP2 accumulated from recent infections [17-20]. In contrast, the *P. vivax *detection component of the ICT combo kit failed to detect 12 of the 13 microscopy positive *P. vivax *samples which had parasite densities varying from 10 to 280 parasites/μL. The only RDT positive *P. vivax *sample had a parasite density >500/μL. The results suggest that the detection limit of the ICT combo kit is ~100/μL for *P. falciparum *and >300/μL for *P. vivax*. This is in good agreement with the WHO RDT Product Testing (Round 1) results where the ICT Malaria Combo Cassette Test (ML02) achieved detection rates at 200/μL of 86.08% and 0% for *P. falciparum *and *P. vivax*, respectively [21]. In the epidemiological setting of Temotu where 65.6% of *P. vivax *infected subjects have parasite densities <100/μL plus a number of sub-microscopic infections, the ICT combo kit is clearly not suitable for detecting *P. vivax *infections.

The Temotu Province is preparing for malaria elimination. The epidemiology data presented here point out clearly that passive and active case detection will not be very useful in identifying infections as majority of them were asymptomatic. Malaria elimination strategies being considered for this island are either some form of active parasite detection programme or mass drug administration (MDA). The first option may take the form of mass screening and treatment of residents of sentinel villages known to have habitual high parasite rates on a regular basis to monitor parasite prevalence. As the majority of infected subjects were observed to carry low and sub-microscopic parasite densities the diagnostic method used to screen will have to be particularly suited to detecting low parasite densities. This is critical for the implementation of screening and treatment interventions, and the monitoring and evaluation of the success of the interventions. The MDA option is controversial and not currently recommended by the WHO.

For case management of *P. falciparum *in this setting, ICT combo kit provided promising results. Several other RDTs that showed comparable panel detection scores at 200/μL in the WHO Product Testing Rounds 1 and 2 [21][22] would be expected to perform at a similar level. However, this study also highlights the high prevalence of infections at <200/μL, indicating an urgent need to test how these top performing RDTs in the WHO Product Testing Programme perform at low parasite densities. This type of information is important for malaria elimination countries considering mass screening and treatment campaigns. It is likely that to detect the large number of sub-microscopic *P. falciparum *infections, a molecular-based assay will be required.

Elimination of *P. vivax *will eventually become the problem due to relapsing cases caused by the activation of hypnozoites in the liver 2-3 years after the initial infection. Level 1 microscopy seems to offer an advantage in detecting this species at very low parasite densities. Again, the incorporation of molecular based PCR detection method was found to detect more *P. vivax *infected subjects. However, the conventional PCR is difficult to perform in the field due to requirements for DNA extraction, sophisticated equipment and reagents. Therefore, a simple, cheap and easy-to-perform molecular assay is required. The deployment of such an assay combined with microscopy or RDT will provide the best chance of detecting the majority of infected individuals. The purpose of using this molecular assay would not be for treating clinical patients, but for finding asymptomatic infections where the individuals can be followed up for treatment on the next day or even longer.

## Conclusion

Results of a mass blood survey conducted in Temotu Province in Solomon Islands showed that a large proportion of *Plasmodium *infected individuals were asymptomatic with low and sub-microscopic parasite densities. With the proposed elimination of malaria from this Province these findings present a challenge for malaria diagnostics in mass screening and treatment programmes and thus for elimination. A combination of methods, or new diagnostics, may be required to detect infections in these asymptomatic parasite reservoirs.

## Competing interests

The authors declare that they have no competing interests.

## Authors' contributions

IH, DS and QC conceived the study. IH carried out microscopy examination, QA, data analysis and drafted the manuscript. WWS performed microscopy QA, PCR and data analysis. LMB, KG and DK performed PCR and data analysis. AB, LB, AV and DS coordinated and planned the survey. KL performed microscopy and QA. MJ coordinated data collating. MLG contributed to data analysis and the writing of manuscript. QC supervised the PCR, analysed data and drafted the manuscript. All authors read and approved the final manuscript.
